# Key role played by mesophyll conductance in limiting carbon assimilation and transpiration of potato under soil water stress

**DOI:** 10.3389/fpls.2024.1500624

**Published:** 2024-12-02

**Authors:** Quentin Beauclaire, Florian Vanden Brande, Bernard Longdoz

**Affiliations:** BIODYNE Biosystems Dynamics and Exchanges, TERRA Teaching and Research Center, Gembloux Agro-Bio Tech, University of Liege, Gembloux, Belgium

**Keywords:** modeling, photosynthesis, stomata, drought, partitioning, potato, mesophyll

## Abstract

**Introduction:**

The identification of the physiological processes limiting carbon assimilation under water stress is crucial for improving model predictions and selecting drought-tolerant varieties. However, the influence of soil water availability on photosynthesis-limiting processes is still not fully understood. This study aimed to investigate the origins of photosynthesis limitations on potato (*Solanum tuberosum*) during a field drought experiment.

**Methods:**

Gas exchange and chlorophyll fluorescence measurements were performed at the leaf level to determine the response of photosynthesis-limiting factors to the decrease in the relative extractable water (REW) in the soil.

**Results:**

Drought induced a two-stage response with first a restriction of CO_2_ diffusion to chloroplasts induced by stomatal closure and a decrease in mesophyll conductance, followed by a decrease in photosynthetic capacities under severe soil water restrictions. Limitation analysis equations were revisited and showed that mesophyll conductance was the most important constraint on carbon and water exchanges regardless of soil water conditions.

**Discussion:**

We provide a calibration of the response of stomatal and non-stomatal factors to REW to improve the representation of drought effects in models. These results emphasize the need to revisit the partitioning methods to unravel the physiological controls on photosynthesis and stomatal conductance under water stress.

## 1 Introduction

European ecosystems are facing more intense and frequent water stress events due to altered rainfall patterns and rising temperatures induced by anthropogenic climate change (Samaniego et al., 2018). Precipitation shortage episodes perturbate plant water status and induce disruptions of the water and carbon cycles through the inhibition of carbon assimilation and transpiration (Bertolino et al., 2019; Fahad et al., 2017; Trenberth et al., 2014). As a result, ecosystem services such as food production and carbon storage are strongly impacted by the lack of soil water (Chang and Bonnette, 2016; Hendrawan et al., 2022; Kang et al., 2021). Land–atmosphere feedbacks originating from the perturbation of such processes may exacerbate climate change through water stress intensification (Anderegg et al., 2019; Hartick et al., 2022). An in-depth understanding of the effects of drought on plant physiology is required to predict future ecosystem service capacities and to improve climate model predictions (Ryu et al., 2019).

Photosynthesis is the process by which plants convert CO_2_ into carbohydrates. Carbon assimilation is mediated by the physiological barriers on the CO_2_ diffusion pathway (i.e., stomatal opening and diffusion within the mesophyll; Gago et al., 2020; Nadal and Flexas, 2018) and by the Rubisco efficiency for fixing CO_2_ in the Calvin cycle (Farquhar et al., 1980). Uncertainties remain on the importance of each limiting factor under soil water-limiting conditions (Rogers et al., 2017).

Quantifying the importance of photosynthesis-limiting factors under drought is also pivotal for assessing phenotype plasticity and selecting drought-tolerant plant species (Lupo and Moshelion, 2024; Nguyen et al., 2023). To that end, mechanistic modeling can be used to disentangle the complexity of the mechanisms regulating plant response to water stress (Stirbet et al., 2020). In the Farquhar–von Caemmerer–Berry (FvCB) model (Farquhar et al., 1980), carbon assimilation under high irradiance ( $A_{sat}$ ) is constrained by stomatal conductance ( ${ɡ}_{s}$ ), mesophyll conductance ( ${ɡ}_{m}$ ), and the maximum carboxylation rate of Rubisco ( $V_{cmax}$ ). The quantitative contribution of each of these factors in limiting photosynthesis under water stress can be estimated by, first, writing the total derivative of $A_{sat}$ as a sum of the total derivative of these factors and, second, by estimating the response of these factors to soil water availability. This method, also known as limitation analysis (Grassi and Magnani, 2005; Jones, 1985), can be used to partition photosynthesis limitations between stomatal (i.e., a decrease in $A_{sat}$ originating from ${ɡ}_{s}$ ) and non-stomatal factors (i.e., a decrease in $A_{sat}$ originating from ${ɡ}_{m}$ and/or $V_{cmax}$ ).

Stomata are the gates of CO_2_ diffusion and water transpiration at the leaf surface. Stomatal opening is regulated by a complex interplay of abiotic and biotic factors. For instance, it is well known that an increase in vapor pressure deficit (VPD) drives the closure of stomata through the evaporation of water in the guard cells (McAdam and Brodribb, 2016). In addition, carbon assimilation regulates stomatal opening to balance the CO_2_ diffusion with the efficiency of the Calvin cycle (Wong et al., 1979). A mechanistic formulation of these relationships was proposed by Cowan and Farquhar (1977), who hypothesized that stomatal opening is regulated to maximize carbon gains and minimize water losses over a constant time interval. This optimization theory is at the basis of the unified stomatal optimality (USO) model where ${ɡ}_{s}$ is expressed as a function of VPD, CO_2_ concentration at the leaf surface, carbon assimilation, and the stomatal sensitivity to photosynthesis ( ${ɡ}_{1}$ ) (Medlyn et al., 2011). This last, which is the slope of the USO model ( ${ɡ}_{1}$ ), is linked to the water use strategy of the plant by being inversely proportional to the marginal carbon cost of water (Medlyn et al., 2011). During drying-up episodes, short timescale variations of ${ɡ}_{1}$ can be used as an indicator of plants’ adaptation strategy. In the framework of the optimality theory, plants can maximize carbon gains (increase in ${ɡ}_{1}$ ), minimize water losses (decrease in ${ɡ}_{1}$ ), or keep the same balance between carbon gains and water losses (constant ${ɡ}_{1}$ ). The response of ${ɡ}_{1}$ to soil water availability is likely species or plant functional type (PFT)-specific (Beauclaire et al., 2023; Gourlez de la Motte et al., 2020; Héroult et al., 2013; Zhou et al., 2013). Although the formulation of the relationship between ${ɡ}_{s}$ and $A_{sat}$ , and the water cost associated with the opening of stomata are still active research topics in the scientific community (Lamour et al., 2022; Mrad et al., 2019), the USO model has become a reference for representing stomatal behavior in land surface models (LSMs) (Kala et al., 2015; Lawrence et al., 2019; Sabot et al., 2022).

As ${ɡ}_{s}$ is mediated by carbon assimilation, a decrease in ${ɡ}_{s}$ can also be induced by biochemical or mesophyll limitations, which regulate stomatal opening with the mesophyll demand for CO_2_ (Lemonnier and Lawson, 2023; Medlyn et al., 2011; Zhou et al., 2013). As a result, ${ɡ}_{s}$ and $A_{sat}\;$ are strongly coupled, and stomatal closure can originate either from an optimal stomatal adaptation or from a disguised effect of mesophyll conductance and/or carboxylation rate of Rubisco (Medlyn et al., 2011; Zhou et al., 2013). This feedback effect complicates the identification of the origins of stomatal closure and photosynthesis limitations under water stress. Using ${ɡ}_{1}\;$ as evidence of optimal stomatal control on photosynthesis theoretically allows to identify the feedback effect of non-stomatal factors on stomatal closure by linking photosynthesis limitations to the stomatal optimality theory (Zhou et al., 2013). As a result, coupling the USO and FvCB models in the limitation analysis would enable a quantitative assessment of the effects of ${ɡ}_{1}$ , VPD, ${ɡ}_{m}$ , and $V_{cmax}$ on ${ɡ}_{s}\;$ and $A_{sat}$ . To our knowledge, this study is the first to develop this approach. The limitations of photosynthesis originating from stomatal closure induced by a decrease in ${ɡ}_{1}\;$ or ${ɡ}_{s}$ are further referred to as a stomatal origin limitation (SOL), while an effect of ${ɡ}_{m}$ and/or $V_{cmax}$ is referred to as a non-stomatal origin limitation (NSOL) (Beauclaire et al., 2023; Gourlez de la Motte et al., 2020).

Soil water content (SWC) is a key eco-hydrological variable impacting plant metabolism and more globally carbon and water fluxes (Zhou et al., 2021). In particular, lack of soil water triggers complex mechanisms which regulate the water flow in the plant to avoid hydraulic failure (Martínez-Vilalta et al., 2014). When soil edaphic proprieties are known, SWC can be used to determine the relative extractable water (REW) for plant uptake (Granier et al., 2007), which is often used in LSMs as a drought index to implement water stress effects on photosynthesis originating from either SOL or NSOL (Vidale et al., 2021). The response of FvCB and USO model parameters to decreasing soil water availability strongly differs across PFTs, which makes REW a critical variable for modeling the response of terrestrial ecosystems to drought (Peters et al., 2018; Rogers et al., 2017; Vidale et al., 2021; Zhou et al., 2013).

Potato is one of the most important crops, providing food for more than one billion people around the world (Lutaladio and Castaldi, 2009). In Europe, more than 400,000 hectares of arable land are used for potato cultivation (Goffart et al., 2022). This crop are highly sensitive to water stress because of its shallow root system and its inability to extract water from deeper soil layers (Obidiegwu, 2015). In particular, tuber bulking is a critical stage of potato growth, as it determines the yield and quality of the harvest (Gervais et al., 2021). Partitioning photosynthesis limitations is crucial for selecting drought-tolerant varieties and ensuring food security. We have implemented this approach during a drought experiment on field-grown potatoes. The goals of this study were i) to describe the response of $A_{sat}$ , ${ɡ}_{s}$ , ${ɡ}_{m}$ , ${ɡ}_{1}$ , and $V_{cmax}$ to the decrease in REW; ii) to perform a limitation analysis on $A_{sat}$ using ${ɡ}_{s}$ or ${ɡ}_{1}$ , ${ɡ}_{m}$ , $V_{cmax}$ , and VPD as explanatory variables; and finally iii) to define REW thresholds from which each of these limitations occurred.

## 2 Material and methods

### 2.1 Plant materials and experimental setup

Potato plants were grown on a 4-ha experimental land located in Belgium, approximately 50 km southeast of Brussels (50°33′47.772″N, 4°42′46.403″E). This cropland is usually used for cultivating chicory, sugar beet, and winter wheat. In total, 88 tubers of potato (*Solanum tuberosum*, cv Agria) were planted under a plastic polytunnel greenhouse 12m long and 5m wide.

SWC and soil temperature were measured using time domain reflectometers (ML3 ThetaProbe, Delta-T Devices Ltd., Cambridge, UK) placed at depths of 10 cm and 30 cm. Air humidity and air temperature were measured using a resistive platinum thermometer and electrical capacitive hygrometer (HMP155, Vaisala Oyj, Helsinki, Finland) placed under the plastic tunnel at 1.5-m height. The tubers were planted on May 15, 2020, and the first leaves appeared on June 4, 2020, which were considered the emergence [i.e., day after emergence (DAE) of 0].

Soil water availability was quantified by calculating the REW of the first soil horizon, where most of the root water uptake of potato is expected to occur (Beauclaire et al., 2023):


(1) $$\begin{array}{c} REW=\{\frac{\theta_{H1}\;-\theta_{wp,H1}\;}{\theta_{fc,H1}-\theta_{wp,H1}} \end{array}$$

where $\theta_{wp,H1}$ = 15.6 and $\theta_{fc,H1}\;$ = 35.01 (cm^3^ cm^−3^) are respectively the wilting point and the field capacity of the first horizon (H_1_: 0–30 cm) and $\theta_{H1}\;$ is the SWC measured in H_1_, which was calculated as the weighted mean of SWC measurements at depths of 10 cm and 30 cm (with a weight of 2/3 and 1/3, respectively). $\theta_{wp,H1}$ and $\theta_{fc,H1}$ were estimated from soil water retention curves using the van Genuchten (VG) model (van Genuchten, 1980). Soil samples were collected before the experiment at a 15cm depth (three replicates) and were saturated for at least 24 h in distilled water. The pressure plate method (Richards, 1948—following the ISO 11274 standard) was applied, and the measurements of the suction head and SWC were recorded. $\theta_{wp,H1}$ and $\theta_{fc,H1}$ were estimated as the SWC at a pF (log of the suction head) of 4.2 and 2.0, respectively. VG model parameters and retention curves of the three soil samples are given in the **Supplementary Material** (**Supplementary Figure S1**).

Over a first period of 35 days, all the plants were hand-watered to ensure that $\theta_{H1}\;$ remained near field capacity. The drought treatment consisted in withholding irrigation to simulate a long-term precipitation deficit on half of the plants. The other half was hand-watered during the experiment. The drought treatment started on DAE 40 (corresponding to the beginning of the tuber bulking stage) and stopped on DAE 74 (corresponding to the appearance of the first signs of senescence on the irrigated plants). All plants experienced the same photosynthetic photon flux density (PPFD) in the photosynthetic active radiation (PAR), temperature, and VPD conditions under the plastic tunnel.

### 2.2 Leaf-level measurements

**Figure 1:**
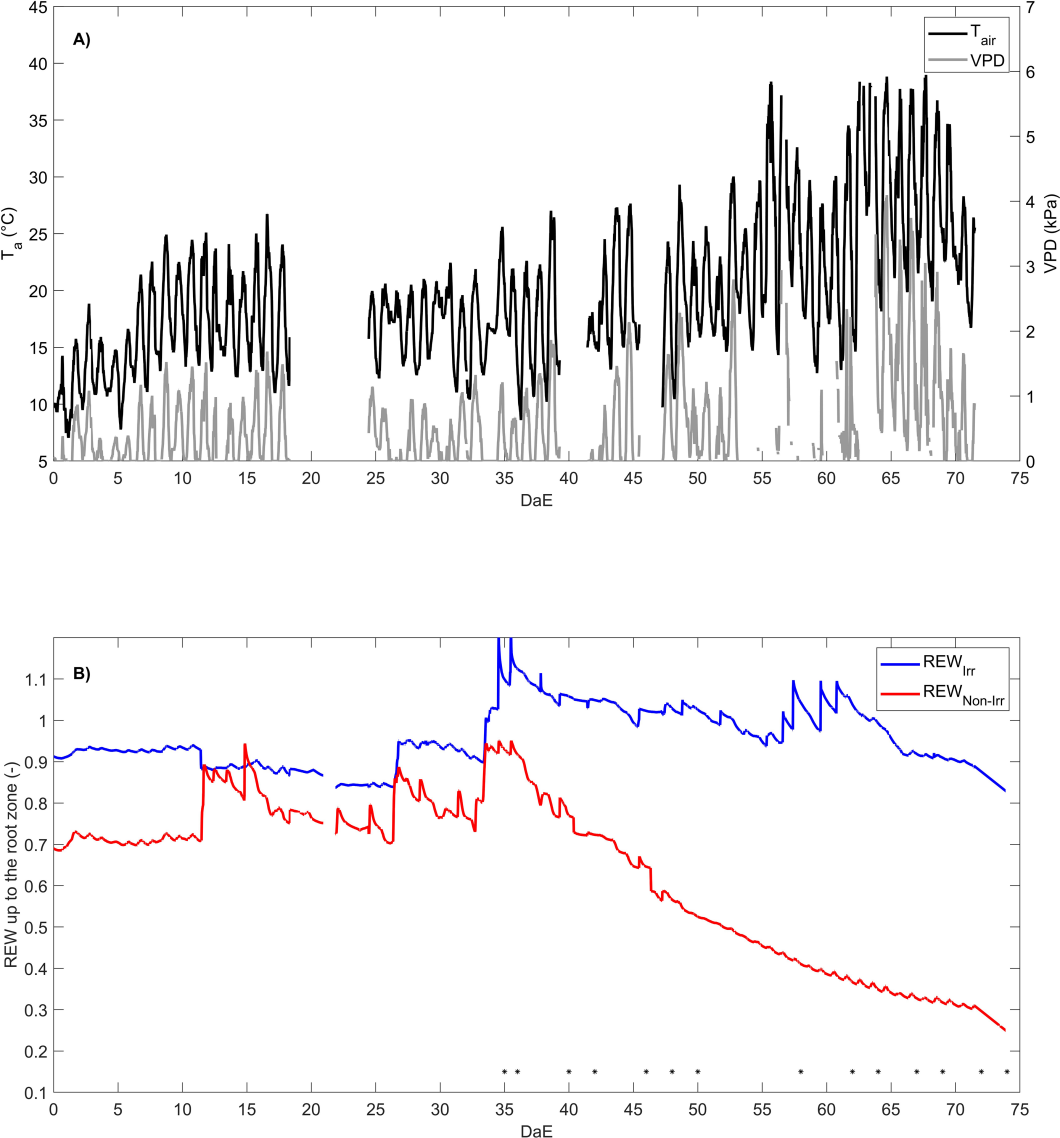
Temporal evolution of air temperature ( $T_{\mathrm{air}}$ ) and air vapor pressure deficit (VPD) under the plastic polytunnel greenhouse **(A)** and relative extractable water (REW) of the irrigated plot ( $REW_{irr}$ ) and non-irrigated plot ( $REW_{non-irr}$ ) **(B)**. The asterisk indicates the days when leaf-level measurements were conducted. DaE is day after emergence.

Gas exchange and chlorophyll fluorescence measurements were conducted during the tuber bulking stage at 14 different dates (between DAE 35 and DAE 74; **Figure 1**) from 10 a.m. to 4 p.m. Only the youngest leaves in the upper part of the plant were selected by randomly sampling irrigated and non-irrigated plants. Measurements were performed using a LI-COR LI-6400 equipped with a LI-6400-40 fluorescence chamber (LI-COR Inc., Lincoln, NE, USA). The following procedure was applied to each leaf sample. The CO_2_ concentration in the chamber ( $C_{s}$ ) was set to 400 μmol mol^−1^, the PPFD in the PAR was set to 1,200 µmol m^−2^ s^−1^, and the air humidity and temperature were maintained at ambient levels. After stabilization of the steady-state fluorescence signal ( $F_{s}$ ), a multiphase flash with a saturation light of 9,000 µmol m^−2^ s^−1^ was applied, and the maximum fluorescence intensity under the light ( $F_{m}^{'})\;$ was measured. In addition, $A_{sat}$ , leaf temperature, stomatal conductance to water vapor ( ${ɡ}_{sw}$ ), CO_2_ concentration in sub-stomatal cavities ( $C_{i}$ ), and the vapor pressure deficit at the leaf surface ( $VPD_{leaf}$ ) were recorded. Stomatal conductance to CO_2_ ( ${ɡ}_{s}$ ) was calculated by dividing ${ɡ}_{sw}$ by 1.6.

#### 2.2.1 NSOL: $V_{cmax}$ and ${ɡ}_{m}$

$V_{cmax}$ was determined using a single measurement of gas exchanges at light saturation (De Kauwe et al., 2016; Wilson et al., 2000):


(2) $$\begin{array}{c} V_{cmax}=A_{sat}\frac{C_{c}+K_{m}}{C_{c}-\Gamma^{*}} \end{array}$$

where $K_{m}$ is the Michaelis–Menten coefficient, $\Gamma^{*}$ the CO_2_ compensation point, and $C_{c}$ the CO_2_ concentration in the chloroplast. Equation 2 is based on a single measurement of CO_2_ assimilation at light saturation instead of using CO_2_-response curves, where $V_{cmax}$ retrieval is impacted by the sensitivity of the fitting method (Miao et al., 2009). Moreover, leaf respiration ( $R_{d}$ ) was neglected, as it is much smaller than $A_{sat}$ (Knauer et al., 2018; Von Caemmerer, 2013). $K_{m}$ and $\Gamma^{*}\;$ were estimated using C3 plant-based temperature response curves (Bernacchi et al., 2001). $C_{c}$ was calculated using the Fick law (Farquhar and Sharkey, 1982):


(3) $$\begin{array}{c} C_{c}=C_{i}-\frac{A_{sat}}{{ɡ}_{m}} \end{array}$$

where ${ɡ}_{m}$ is determined using the “variable electron transport” method (Harley et al., 1992):


(4) $$\begin{array}{c} {ɡ}_{m}=\frac{A_{sat}}{C_{i}-\frac{\Gamma^{*}\;\left(J_{F}+8A_{sat}\right)}{J_{F}-4A_{sat}}} \end{array}$$

where $J_{F}$ is the electron transport rate estimated from PPFD, α the leaf absorptance in the PAR, $\varphi_{PSII}$ the photochemical efficiency of PSII open centers, and $\beta_{PSII}$ the fraction of the absorbed PAR allocated to PSII (Genty et al., 1989; Valentini et al., 1995):


(5) $$\begin{array}{c} J_{F}=\alpha \cdot \beta_{PSII}\cdot \varphi_{PSII}\cdot PPFD \end{array}$$

In Equation 5, $\varphi_{PSII}$ was determined from $F_{m}^{\prime }$ and $F_{s}$ (Kramer et al., 2004):


(6) $$\begin{array}{c} \varphi_{PSII}=\frac{F_{m}^{\prime }-F_{s}}{F_{m}^{\prime }} \end{array}$$

and $\alpha \cdot \beta_{PSII}$ was determined from the linear relationship between $\varphi_{PSII}$ and the apparent quantum efficiency of the linear electron transport $\varphi_{e-}$ (Valentini et al., 1995):


(7) $$\begin{array}{c} \alpha \cdot \beta_{PSII}=\frac{4}{k} \end{array}$$

where 4 is the number of electrons needed per CO_2_ molecule fixed and k the slope of the linear relationship between $\;\varphi_{e-}$ and $\;\varphi_{PSII}$ . Under non-photorespiratory conditions, $\;\varphi_{e-}$ can be estimated by the apparent quantum efficiency of CO_2_ uptake $\;\varphi_{CO2}$ , which is obtained by dividing the net CO_2_ assimilation by the incident PAR (Genty et al., 1989). Non-photorespiratory conditions were set by adding pure N_2_ (1% O_2_) into the LI-COR LI-6400 chamber. The meteorological conditions were maintained at ambient levels, and the incoming PPFD was set to the following values: 2,000, 1,500, 1,200, 1,000, 800, 600, 400, 200, 100, and 0 µmol m^−2^ s^−1^. Gas exchanges and fluorescence intensities were measured for each PPFD value. $\;\varphi_{CO2}$ was calculated as the ratio of net carbon assimilation to PAR. The slope of fitted linear relationship between $\varphi_{CO2}$ and $\varphi_{e-}$ ( k ) was used to determine $\alpha \cdot \beta_{PSII}$ using Equation 7. These measurements were conducted on three leaf samples for irrigated and non-irrigated plants and were repeated three times during the drought treatment (i.e., DAEs 42, 64, and 73).

#### 2.2.2 SOL: *g_s_* and *g_1_*

In the USO model, ${ɡ}_{s}$ is a function of $VPD_{leaf}$ , $C_{s}$ , and $A_{sat}$ (Medlyn et al., 2011):


(8) $$\begin{array}{c} {ɡ}_{s}=\left(1+\frac{{ɡ}_{1}}{\sqrt{VPD_{leaf}}}\right)\frac{A_{sat}}{C_{s}} \end{array}$$

where the minimum stomatal conductance is neglected under high irradiance (Medlyn et al., 2017), and ${ɡ}_{1}$ is the stomatal sensitivity to photosynthesis, which is inversely related to the marginal water use efficiency (WUE) (Medlyn et al., 2011). ${ɡ}_{1}\;$ can be determined by combining the Fick law describing the CO_2_ diffusion through stomata with Equation 8, which gives (Medlyn et al., 2017)


(9) $$\begin{array}{c} {ɡ}_{1}=\frac{\frac{C_{i}}{C_{s}}\sqrt{VPD_{leaf}}}{1-\frac{C_{i}}{C_{s}}} \end{array}$$

### 2.3 Statistical analysis

${ɡ}_{m}$ values were discarded when $C_{i}$ was outside of the range 150–350 μmol mol^−1^, which minimizes errors in $R_{d}$ and Γ* and by extension in ${ɡ}_{m}$ (Harley et al., 1992; Niinemets et al., 2006; Veromann-Jürgenson et al., 2017). Moreover, $V_{cmax}\;$ and ${ɡ}_{m}$ were normalized at 25°C ( $V_{cmax,25}$ , ${ɡ}_{m,25}$ ) using the Arrhenius temperature response function parameterized on tobacco (Bernacchi et al., 2002, 2001). Gas exchange and chlorophyll fluorescence-related variables (i.e., $A_{sat}$ , ${ɡ}_{s}$ , ${ɡ}_{m,25}$ , ${ɡ}_{1}$ , and $V_{cmax,25}$ ) were averaged for each day of measurement and drought treatment (irrigated and non-irrigated), thus regrouping measurements performed under similar meteorological and edaphic conditions.

The response of $A_{sat}$ , ${ɡ}_{s}$ , ${ɡ}_{m,25}$ , ${ɡ}_{1}$ , and $V_{cmax,25}$ to the decrease in REW was assessed using a linear-plateau model, which consists in a constant value ( $y_{max}$ ) and a linear segment (with slope a and intercept b ) on either side of a threshold ( $REW_{th}$ ). Such model has already been used to describe the response of SOL and NSOL to soil water availability of potato crops at the ecosystem scale (Beauclaire et al., 2023) and is used to implement the response of LSM parameters to drought (Vidale et al., 2021). The statistical significance of the linear-plateau model was assessed by comparing its Akaike information criterion corrected for low sample size (AICc; Burnham et al., 2002) to the one of a higher parsimonious model (i.e., a linear model with one slope and intercept). The model with the lowest AICc explains the greatest amount of variation while being the more parsimonious (Burnham et al., 2011; Scoffoni et al., 2012). Differences between models were considered meaningful when their AICcs differed by at least 7 (Burnham et al., 2011). If the difference was less than 7, the segmented model was selected, as such a relationship has already been observed for potato (Beauclaire et al., 2023). Model performance was assessed using the coefficient of determination (R^2^) and the standard deviation (SD) of fitted parameters. The segmented regression was fitted using the “nlsm” function from the “nlraa” package in R Studio (Archontoulis and Miguez, 2015; Miguez, 2023). Statistical difference between $REW_{th}$ parameters was tested by calculating the p-value of a t-test using the fitted values and their corresponding standard deviation (Clogg et al., 1995; Paternoster et al., 1998).

### 2.4 Limitation analysis

The first limitation scheme used in this study was proposed by Jones (1985), where SOL was associated to a decrease in ${ɡ}_{s}$ caused by a decrease in either $V_{cmax}$ or ${ɡ}_{m}$ . The relative variation of $A_{sat}$ compared to its maximum value $\frac{dA_{sat}}{A_{sat}}$ is written as the sum of the relative variations of ${ɡ}_{s}$ , ${ɡ}_{m}$ , and $V_{cmax}$ , as follows (Grassi and Magnani, 2005; Jones, 1985):


(10) $$\begin{array}{c} \frac{dA_{sat}}{A_{sat}}=\frac{d{ɡ}_{s}}{{ɡ}_{s}}l_{ɡs}+\frac{d{ɡ}_{m}}{{ɡ}_{m}}l_{ɡm}+\frac{dV_{cmax}}{V_{cmax}}l_{Vcmax}=L_{ɡs}+L_{ɡm}+L_{Vcmax} \end{array}$$

(11) $$\begin{array}{c} l_{ɡs}=\frac{\frac{{ɡ}_{t}}{{ɡ}_{s}}\frac{\delta A_{sat}}{\delta C_{c}}}{{ɡ}_{t}+\frac{\delta A_{sat}}{\delta C_{c}}} \end{array}$$

(12) $$\begin{array}{c} l_{ɡm}=\frac{\frac{{ɡ}_{t}}{{ɡ}_{m}}\frac{\delta A_{sat}}{\delta C_{c}}}{{ɡ}_{t}+\frac{\delta A_{sat}}{\delta C_{c}}} \end{array}$$

(13) $$\begin{array}{c} l_{Vcmax}=\frac{{ɡ}_{t}}{{ɡ}_{t}+\frac{\delta A_{sat}}{\delta C_{c}}} \end{array}$$

where $l_{ɡs}$ , $l_{ɡm}$ , and $l_{Vcmax}$ are respectively the relative stomatal, mesophyll, and biochemical limitations (corresponding to dimensionless quantity between 0 and 1 that gives the proportion of the total limitation), and $L_{ɡs},L_{ɡm}\;$ , and $L_{Vcmax}$ are the contributions of respectively the stomatal, mesophyll, and biochemical limitations to the relative variation of $A_{sat}$ . ${ɡ}_{t}$ is the total conductance to CO_2_ diffusion ( ${ɡ}_{t}^{-1}={ɡ}_{s}^{-1}+{ɡ}_{m}^{-1}$ ), and $\delta A_{sat}/\delta C_{c}$ is the partial derivative of $\;A_{sat}$ with respect to $C_{c}$ calculated using Equation 2. In this study, Equation 10 was normalized by $dA_{sat}/A_{sat}\;$ to improve the interpretation of the data. The temporal dynamics of these relative variations can be explained solely by REW and VPD, as the relationship to temperature was already considered by normalizing $V_{cmax}$ and ${ɡ}_{m}$ at 25°C, as well as the one to solar radiation by collecting the data at light saturation.

This approach has two drawbacks. First, the decrease in $A_{sat}$ originating from stomatal closure through a decrease in ${ɡ}_{s}$ can be induced by ${ɡ}_{m}$ and $V_{cmax}$ , which may result in the underestimation of the contribution of non-stomatal factors in limiting photosynthesis. Second, identifying the contribution of REW to the variation in $L_{ɡs}$ is complex, as VPD has varied during the experiment. To tackle this issue, we used ${ɡ}_{1}$ instead of ${ɡ}_{s}$ as SOL. This allows first, to separate the feedback effect of NSOL on stomatal conductance and, second, to consider the effect of VPD on stomatal closure (Zhou et al., 2013). As a result, Equation 10 was modified by calculating the total derivative of ${ɡ}_{s}\;$ using the USO model, which gives (derived in **Supplementary Method S1**):


(14) $$\begin{array}{c} \frac{dA_{sat}}{A_{sat}}=\frac{d{ɡ}_{1}}{{ɡ}_{1}}\left(\frac{l_{ɡs}}{1-l_{ɡs}}\frac{C_{i}}{C_{s}}\right)+\frac{d{ɡ}_{m}}{{ɡ}_{m}}\left(\frac{l_{ɡm}}{1-l_{ɡs}}\right)+\frac{dV_{cmax}}{V_{cmax}}\left(\frac{l_{Vcmax}}{1-l_{ɡs}}\right)-\left(\frac{1}{2}\frac{l_{ɡs}}{1-l_{ɡs}}\frac{C_{i}}{C_{s}}\right)\frac{dVPD}{VPD} \end{array}$$

(15) $$\begin{array}{c} \frac{dA_{sat}}{A_{sat}}=\frac{d{ɡ}_{1}}{{ɡ}_{1}}l_{ɡ1,USO}+\frac{d{ɡ}_{m}}{{ɡ}_{m}}l_{ɡm,USO}+\frac{dV_{cmax}}{V_{cmax}}l_{Vcmax,USO}+l_{VPD,USO}\frac{dVPD}{VPD} \end{array}$$

(16) $$\begin{array}{c} \frac{dA_{sat}}{A_{sat}}=L_{ɡ1,USO}+L_{ɡm,USO}+L_{Vcmax,USO}+L_{VPD,USO} \end{array}$$

where $L_{ɡ1,USO}$ , $L_{ɡm,USO}$ , $L_{Vcmax,USO}$ , and $L_{VPD,USO}$ are the contributions of respectively the optimal stomatal, mesophyll, biochemical, and VPD limitations to the relative variation of $A_{sat}$ using the USO model of stomatal conductance. Equations 15, 16 show that $dA_{sat}/A_{sat}$ can be written as the sum of the relative variations of ${ɡ}_{1}$ , ${ɡ}_{m}$ , $V_{cmax}$ , and VPD. Combining Equations 16, 10 allows to identify the effect of ${ɡ}_{m}$ , $V_{cmax}$ , ${ɡ}_{1}$ , and VPD on the contribution of stomatal closure to photosynthesis, as follows:


(17) $$\begin{array}{c} L_{ɡs}=L_{ɡm,USO}-L_{ɡm}+L_{Vcmax,USO}-L_{Vcmax}+L_{VPD,USO}+\;L_{ɡ1,USO} \end{array}$$

$L_{ɡm,USO}-L_{ɡm}+L_{Vcmax,USO}-L_{Vcmax}$ is the effect of NSOL on stomatal closure, while $L_{VPD,USO}+\;L_{ɡ1,USO}$ is the effect of VPD and ${ɡ}_{1}$ on stomatal closure according to the USO model. The relative variations in Equations 10, 15 are calculated from the difference between the value of the variable at a specific REW and the asymptote of the linear-plateau using $dy/y=\left(y_{max}-y\right)/\left(y_{max}-min\left(y\right)\right)$ , with y being the ordinate at a specific REW value and $y_{max}$ the plateau of the segmented regression. In a similar fashion, dVPD/VPD is determined from the VPD–REW relationship. During precipitation shortage episodes, this relationship is decreasing (i.e., increase in VPD when REW decrease), which was observed during the experiment (**Supplementary Figure S2**). This relationship was confirmed by the data of the nearby eddy covariance station of Lonzée for similar edaphic proprieties (data not shown). Therefore, this linear relationship was used to determine dVPD/VPD at each REW value.

In Equation 14, the ratio $C_{i}/C_{s}$ also plays an important role in the limitation analysis, as it directly influences $L_{ɡ1,USO}$ and $L_{VPD,USO}$ . Using ${ɡ}_{1}$ as SOL implies that any stomatal constraint on $A_{sat}$ should be associated with an increase of the ratio $A_{sat}/{ɡ}_{s}$ . Indeed, following the USO model framework, this constraint corresponds to a maximization of photosynthesis while minimizing water losses. As ${ɡ}_{s}$ and $A_{sat}$ both regulate CO_2_ diffusion through stomatal apertures and CO_2_ fixation in the chloroplasts, the increase in $A_{sat}/{ɡ}_{s}$ is linked to a decrease in $C_{i}/C_{s}$ , illustrating an optimal stomatal control ( $C_{i}/C_{s}∼1-A_{sat}/{ɡ}_{s}$ ). The relationship between $C_{i}/C_{s}$ and REW was also evaluated by fitting a linear-plateau model as described in section 2.4. Note that $L_{VPD,\;USO}$ is per essence negative because of the partial derivative of VPD with respect to $A_{sat}$ , as they are inversely related (i.e., VPD at the denominator in the USO model; Equation 8). As a result, any increase in VPD induces a closure of stomata and a decrease in $A_{sat}$ . A decrease in $V_{cmax}$ , ${ɡ}_{m}$ , or ${ɡ}_{1}$ induces a decrease in $A_{sat}$ for all the other terms of Equation 16.

## 3 Results

### 3.1 Meteorological and edaphic conditions

The decline in soil water availability was synchronized with a period of progressive increase in VPD and air temperature under the plastic polytunnel greenhouse (**Figure 1A**) up to a maximum value of 4.10 kPa and 39.02°C, respectively (**Figure 1A**). Both irrigated and non-irrigated plants faced an increase in atmospheric dryness and air temperature. The REW of the non-irrigated plants decreased after stopping the irrigation and reached 0.24 at the end of the experiment, while the REW of the irrigated plants remained higher than 0.83 due to continuous hand watering (**Figure 1B**).

### 3.2 Response of gas exchanges and chlorophyll fluorescence to drought

**Figure 2:**
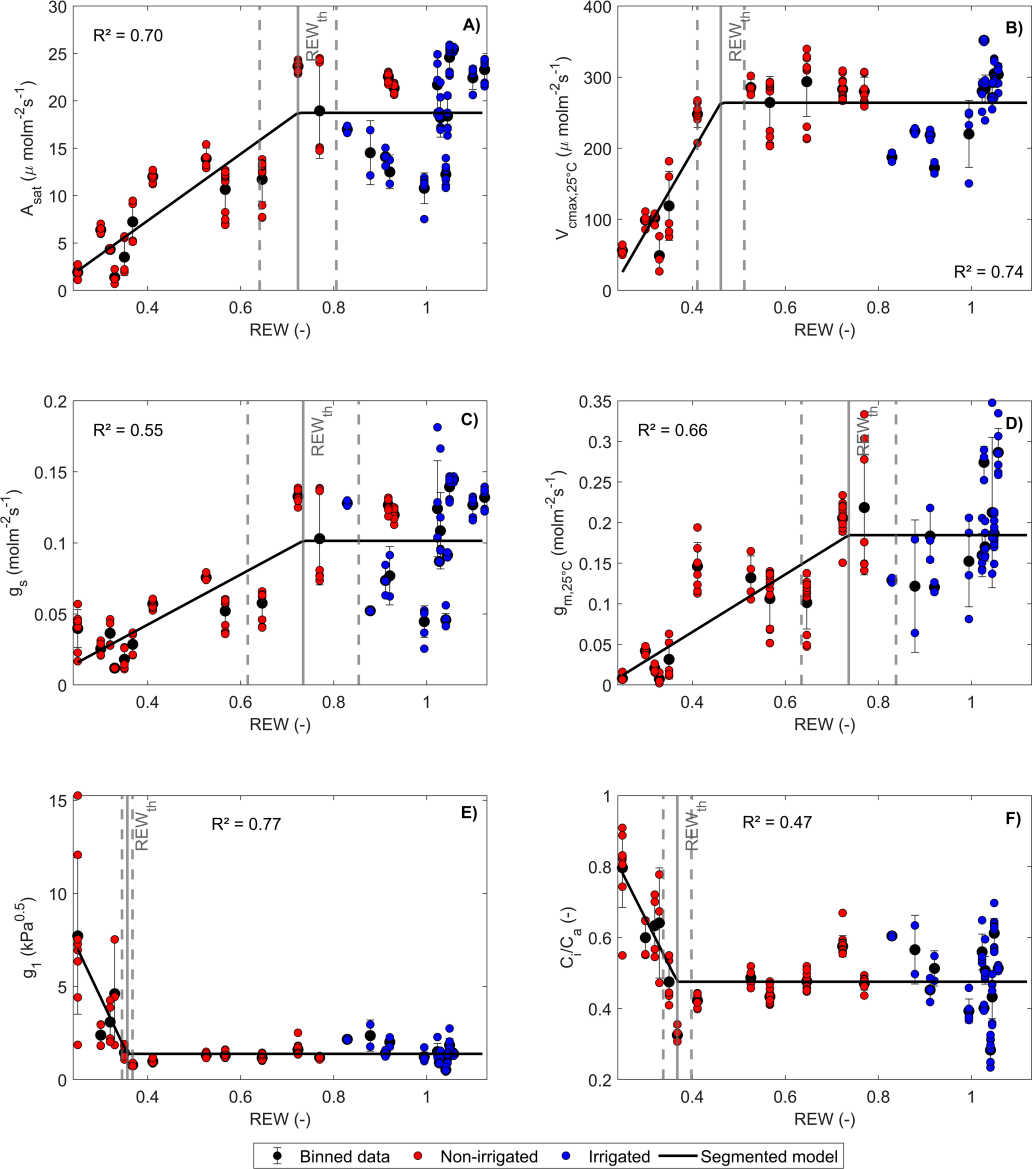
Response of $A_{sat}$ **(A)**, $V_{cmax,25}$ **(B)**, ${ɡ}_{s}$ **(C)**, ${ɡ}_{m,25}$ **(D)**, ${ɡ}_{1}$ **(E)**, and $C_{i}/C_{s}$ **(F)** to relative extractable water (REW). Red and blue dots indicate respectively non-irrigated and irrigated potato plants. The fitted curve represents the linear-plateau regression $y=\{\begin{array}{c} y_{max},\;\;REW>REW_{th}\\ aREW+b,\;\;REW\leq REW_{th} \end{array}$ . Binned data are shown with the corresponding standard deviation (SD). The gray vertical lines indicate $REW_{th}$ ± SD.

$\alpha \cdot \beta_{PSII}$ was not significantly different between irrigated and non-irrigated leaf samples at each DAE and during the experiment (**Supplementary Figure S3**). Therefore, the mean of all $\alpha \cdot \beta_{PSII}$ measurements was used in Equation 5 (i.e., $\text{α}\cdot \beta_{PSII}$ = 0.73 ± 0.08). The linear-plateau model had the lowest AICc compared to the linear model for representing the dependence of $V_{cmax,25}$ , ${ɡ}_{1}$ , and $C_{i}/C_{s}$ on REW. For ${ɡ}_{s}$ , ${ɡ}_{m,25}$ , and $A_{sat}$ , the difference between the AICc of the segmented and the linear model was less than 7 (**Supplementary Table S1**). Therefore, these differences were not considered significant, and the segmented model was chosen for reproducing the response of $A_{sat}$ , $C_{i}/C_{s}$ , ${ɡ}_{s}$ , ${ɡ}_{m,25}$ , and ${ɡ}_{1}$ to REW (**Figure 2**, **Table 1**). The REW thresholds at which $A_{sat}$ , ${ɡ}_{s}$ , and ${ɡ}_{m,25}$ started to decrease were higher than those of $V_{cmax,25\;}$ , ${ɡ}_{1}$ , and $C_{i}/C_{s}$ (**Figure 2**, **Table 1**), which is confirmed by the p-values of the tests comparing these parameters (**Table 2**). Overall, CO_2_ diffusion factors (i.e., ${ɡ}_{m,25}$ and ${ɡ}_{s}$ ) were the first variables to decrease with REW, while biochemical factors (i.e., $V_{cmax,25}$ ) were only impacted by severe REW restrictions. Because of a non-significant difference, the REW thresholds for ${ɡ}_{m,25}$ and ${ɡ}_{s}$ were averaged, corresponding to $REW_{th,ɡs,ɡm}=$ 0.72 ± 0.12. Biochemical limitation ( $V_{cmax,25\;}$ ) was only negatively impacted by severe soil water restrictions ( $REW_{th,Vcmax}=$ 0.43 ± 0.04). ${ɡ}_{1}$ and $C_{i}/C_{s}\;$ increased from a smaller REW threshold compared to $V_{cmax,25\;}$ ( $REW_{th,\;ɡ1,CiCs}=$ 0.37 ± 0.02; **Figure 2**, **Tables 1**, **2**).

**Table 1:** Statistics of the segmented linear regression for the response of *A_sat_*, *V_cmax,25_*, *g_s_*, *g_m,25_*, *g_1_*, *C_i_/C_s_* and to REW.

	*A_sat_* (µmol m^−2^ s^−1^)	*V_cmax,25_* (µmol m^−2^ s^−1^)	*ɡ_s_* (mol m^−2^ s^−1^)	*ɡ_m,25_* (mol m^-2^ s^-1^)	*ɡ_1_* (kPa^0.5^)	*C_i_/C_s_* (-)
$y=\{\begin{array}{c} y_{max},\;x>REW_{th}\\ ax+b,\;x\leq REW_{th} \end{array}$						
*Y_max_* (± *SD*)	18.74 ± 1.00	264.02 ± 11.63	0.10 ± 0.01	0.15 ± 0.01	1.39 ± 0.17	0.48 ± 0.02
α (± *SD*)	35.22 ± 8.38	1125.2 ± 387.6	0.18 ± 0.06	0.29 ± 0.11	−52.67 ± 9.83	−2.59 ± 1.12
*b* (± *SD*)	−6.73 ± 3.86	−255.2 ± 127.9	−0.03 ± 0.03	−0.06 ± 0.05	20.15 ± 3.06	1.43 ± 0.35
*REW_th_* (± *SD*)	0.72 ± 0.08	0.43 ± 0.04	0.73 ± 0.12	0.72 ± 0.13	0.36 ± 0.01	0.37 ± 0.03
R^2^	0.70	0.74	0.55	0.66	0.77	0.47

Parameters are given with their standard deviation (SD).

REW, relative extractable water.

**Table 2:** p-Value of the t-test comparing $REW_{th}$ between $A_{sat}$ , $V_{cmax,25}$ , ${ɡ}_{s}$ , ${ɡ}_{m,25}$ , ${ɡ}_{1}$ , and $C_{i}/C_{s}$ .

p-Value $REW_{th}$	$A_{sat}$	$V_{cmax,25}$	${ɡ}_{s}$	${ɡ}_{m,25}$	${ɡ}_{1}$	$C_{i}/C_{s}$
$A_{sat}$	–					
$V_{cmax,25}$	0.000^***^	–				
${ɡ}_{s}$	0.89^ns^	0.000^***^	–			
${ɡ}_{m,25}$	0.69^ns^	0.000^***^	0.89^ns^	–		
${ɡ}_{1}$	0.00^***^	0.000^***^	0.000^***^	0.000^***^	–	
$C_{i}/C_{s}$	0.00^***^	0.000^***^	0.000^***^	0.000^***^	0.05^ns^	–

*** indicates when the p-value is <0.001 and ns when >0.05.

### 3.3 Limitation analysis

**Figure 3:**
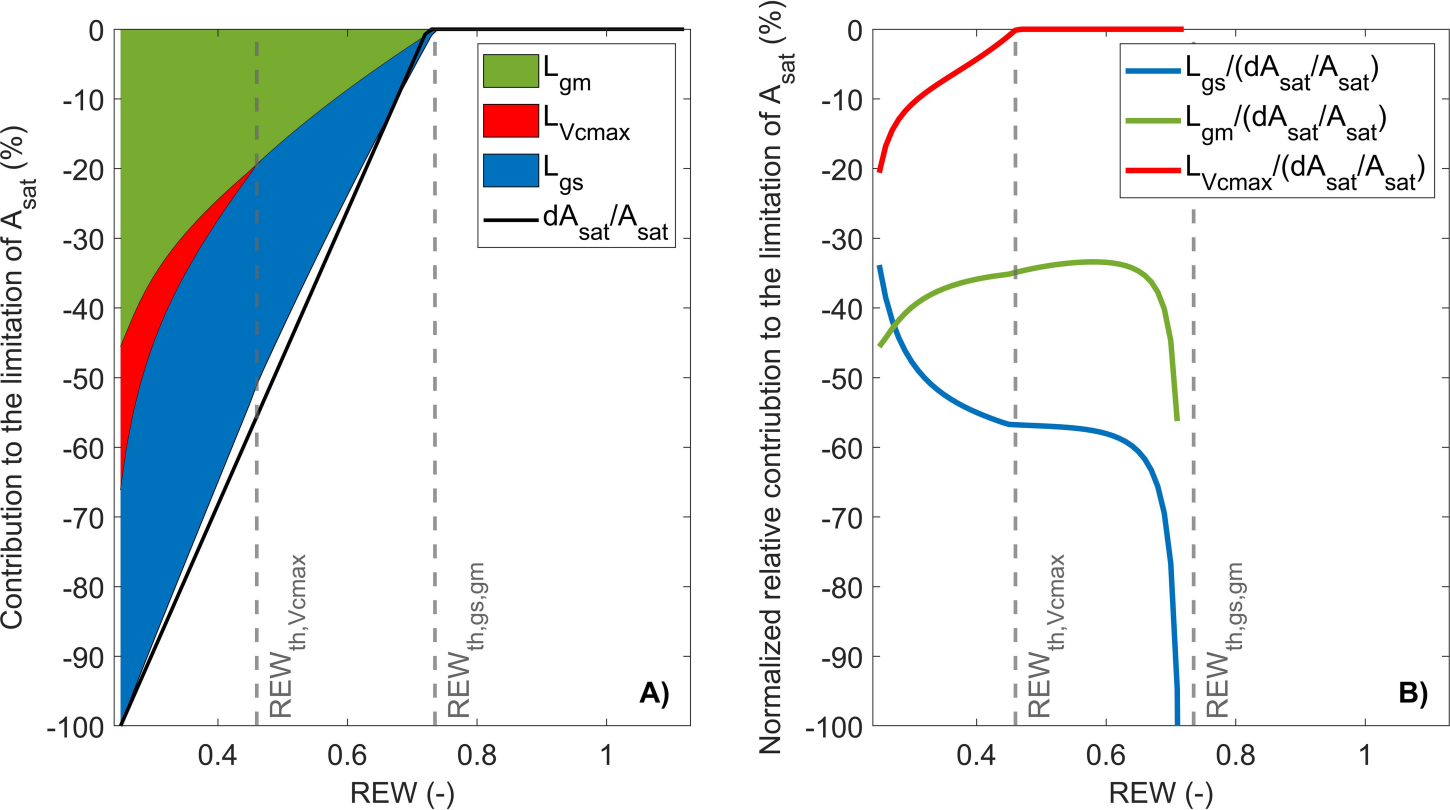
Partitioning of $A_{sat}\;$ limitations between $L_{ɡs}$ , $L_{ɡm}$ , and $L_{Vcmax}$ in response to relative extractable water (REW) with stacked limitation curves **(A)** and normalized relative contribution curves **(B)**. The black line is the relative variation of $A_{sat}$ compared to its maximum value (i.e., $y_{max}$ of the linear-plateau regression), and the gray vertical lines indicate $REW_{th}$ ± SD.

The first limitation analysis scheme used in this study consists in partitioning photosynthesis limitations under high irradiance between $L_{ɡm}$ , $L_{Vcmax}$ , and $L_{ɡs}$ (Jones, 1985). $L_{ɡs}$ was always higher than $L_{ɡm}$ above REW ~ 0.28 (**Figure 3A**), where $L_{ɡm}$ became predominant over $L_{ɡs}$ (i.e., intersection of $L_{ɡs}$ and $L_{ɡm}$ ; **Figure 3B**). When REW was minimum, 34% of the decrease in $A_{sat}$ was explained by $L_{ɡs}$ , 20% by $L_{Vcmax}$ , and 56% by $L_{ɡm}$ (**Figures 3A, B**).

**Figure 4:**
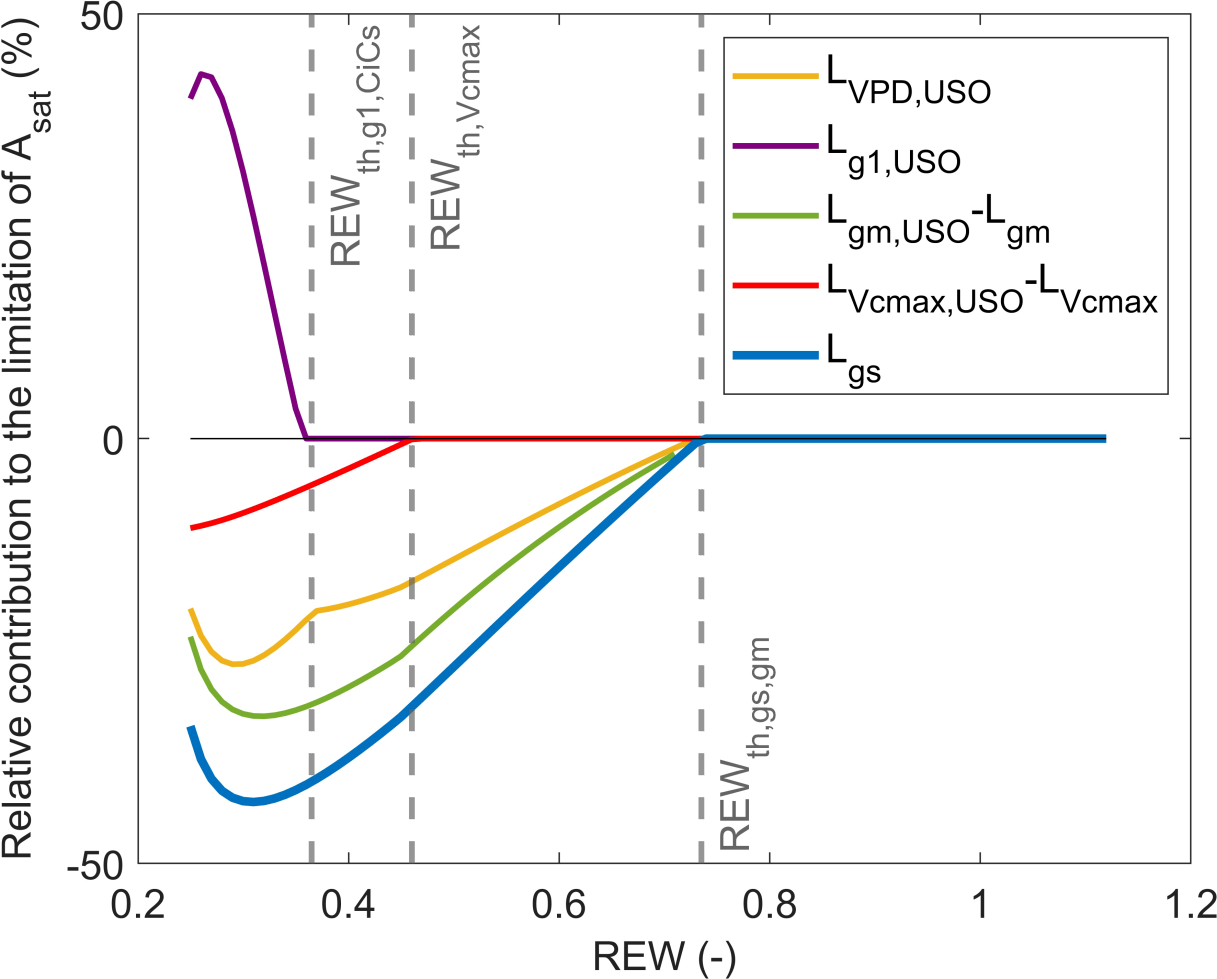
Partitioning of the limiting component induced by stomatal closure ( $L_{ɡs}$ ) into $L_{ɡ1,USO}$ , $L_{ɡm,USO}-L_{ɡm}$ , $L_{Vcmax,USO}-L_{Vcmax}$ , and $L_{VPD,USO}$ in response to relative extractable water (REW). The gray vertical lines indicate $REW_{th}$ ± SD.

**Figure 5:**
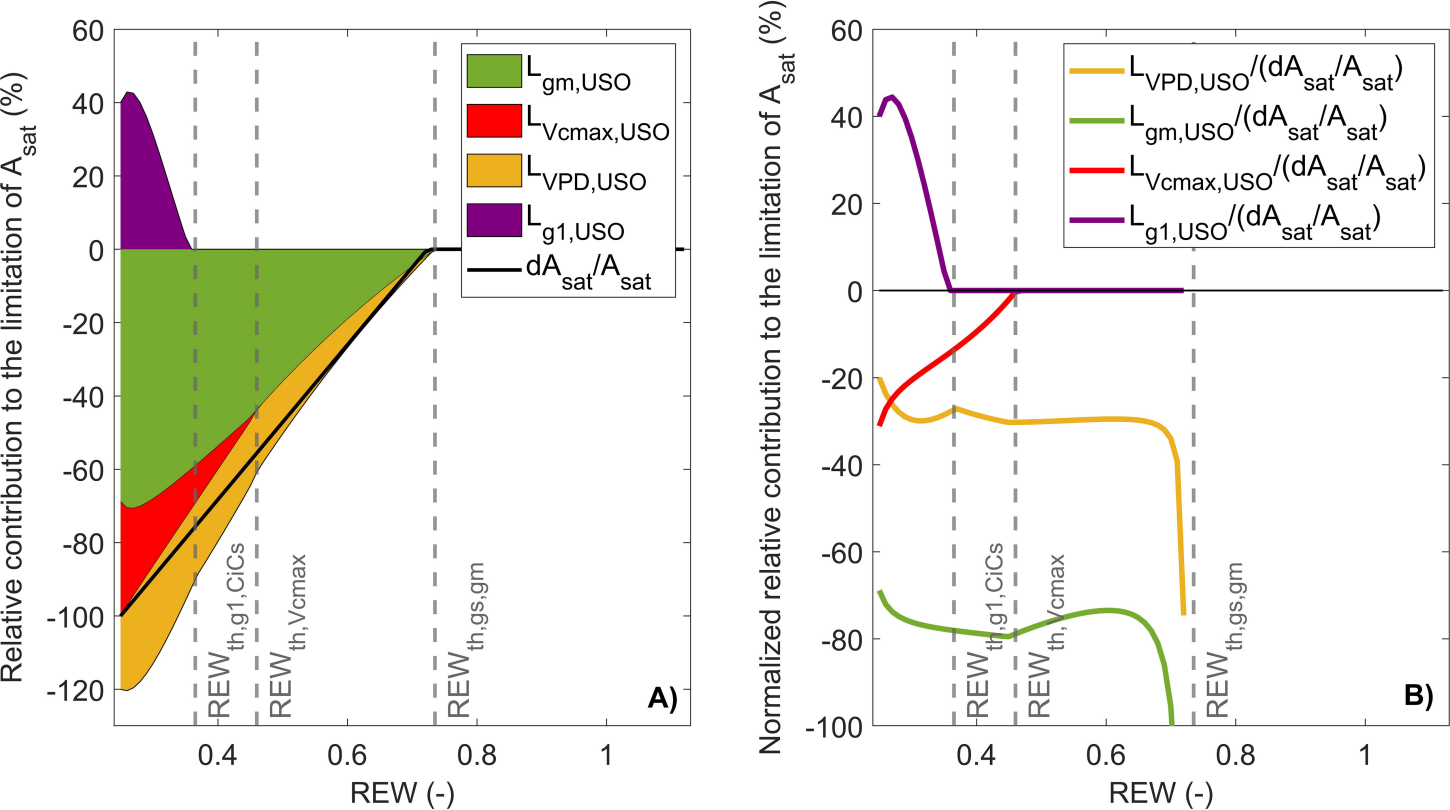
Partitioning of $A_{sat}\;$ limitations between $L_{ɡ1,USO}$ , $L_{ɡm,USO}$ , $L_{Vcmax,USO}$ , and $L_{VPD,USO}$ in response to relative extractable water (REW) with stacked limitation curves **(A)** and shows normalized relative contribution curves **(B)**. The black line is the relative variation of $A_{sat}$ compared to its maximum value (i.e., $y_{max}$ of the linear-plateau regression), and the gray vertical lines indicate $REW_{th}$ ± SD.

This limitation scheme indicated that CO_2_ diffusion factors (i.e., $L_{ɡm}$ and $L_{ɡs}$ ) explained most of the decrease in $A_{sat}$ with a similar contribution. However, using ${ɡ}_{s}$ in the partitioning analysis does not allow to fully identify the origin of the early stomatal closure, as ${ɡ}_{s}$ itself can be influenced by $V_{cmax}$ and ${ɡ}_{m}$ through $A_{sat}$ (Equation 8). This hypothesis is supported by the similar REW threshold for ${ɡ}_{s}$ and ${ɡ}_{m,25}$ , which suggests that the two variables are closely related. Combining Equations 16, 10 showed that the increase in $L_{ɡs}\;$ is mostly caused by ${ɡ}_{m}$ and VPD notably under mild soil water conditions ( $REW>REW_{th,CiCs},\;$ ; **Figure 4**). In particular, $L_{ɡm,USO}$ was always higher than $L_{VPD,USO}$ and $L_{Vcmax,USO}$ (**Figure 4**). Moreover, ${ɡ}_{1}$ had a positive contribution to $L_{ɡs}$ (**Figure 4**), which indicates that the increase in ${ɡ}_{1}$ (**Figure 2E**) promoted the opening of stomata to sustain CO_2_ diffusion to the fixation sites. Once the USO model has been integrated in the limitation analysis on $A_{sat}$ , it can be shown that $L_{ɡm,USO}$ was predominant over $\;L_{VPD,USO}$ regardless of soil water conditions (**Figure 5A**). When REW was minimum, 69% of the decrease in $A_{sat}$ was explained by $L_{ɡm,USO}$ , 31% by $L_{Vcmax,USO}$ , and 20% by $L_{VPD,USO}$ (**Figure 5A**). In these conditions, $L_{ɡ1,USO}$ was positive and reached 40%. The positive contribution of ${ɡ}_{1}$ can be explained by the increase in $d{ɡ}_{1}/{ɡ}_{1}$ (**Figure 2E**), which resulted in an increase in $L_{ɡ1,USO}$ (Equation 15). Such increase in $L_{ɡ1,USO}$ was observed from $REW_{th,\;ɡ1,CiCs}\;$ (**Tables 1**, **2**), which corresponded to low $A_{sat}$ (6.8 μmol m^−2^ s^−1^) and ${ɡ}_{s}$ (0.04 mol m^−2^ s^−1^). Note that the sum of all curves in **Figure 5B** may not necessarily equal 1, as the sum of limiting components when using the USO partitioning scheme did not exactly correspond to $dA_{sat}/A_{sat}$ because of the uncertainties associated with the fitting of the linear-plateau segmented model on measurements (**Figures 2**, **5A**).

## 4 Discussion

The determination of thresholds of soil water availability impacting CO_2_ assimilation is pivotal for calibrating the response of photosynthesis model parameters during drying-up episodes (Vidale et al., 2021). The results of this study showed that soil water-limiting conditions induced a two-stage response of potato to water stress, with ${ɡ}_{s}$ and ${ɡ}_{m}$ being the first variables impacted by the decrease in REW followed by biochemical limitations through the decrease in $V_{cmax}$ . In addition, we used a new partitioning scheme where the total derivative of ${ɡ}_{s}$ was written as a function of its explanatory variables in the USO model (i.e., ${ɡ}_{1}$ , $V_{cmax}$ , VPD , and $A_{sat}$ ). This method allowed to quantify the origins of the decrease in $A_{sat}$ in response to changes in ${ɡ}_{m}$ , $V_{cmax}$ , ${ɡ}_{1}$ , and VPD. This partitioning was compared to the original formulation of photosynthesis limitations of Jones (1985), which attributed the origins of the reduction of $A_{sat}$ to the relative variations of ${ɡ}_{m}$ , $V_{cmax}$ , and ${ɡ}_{s}$ . The comparison between the two schemes provides an estimation of the importance of the factors influencing ${ɡ}_{s}$ and $A_{sat}$ .

### 4.1 Predominance of CO_2_ diffusion constraints on photosynthesis

Stomatal closure is a well-known mechanism of potato to reduce transpiration under water stress (Gerhards et al., 2016; Gordon et al., 1997; Obidiegwu, 2015; Romero et al., 2017; Vos and Oyarzún, 1987). Stomatal closure dynamics are complex and can be directly caused by the evaporation of the water held by guard cells or by the loss of turgor pressure induced by sensing of signaling molecules (Bharath et al., 2021; Ding and Chaumont, 2020; Obidiegwu, 2015; Pirasteh-Anosheh et al., 2016; Zhang et al., 2022). These mechanisms are likely to be synchronized with those influencing mesophyll conductance as evidenced by a similar REW threshold for ${ɡ}_{s}$ and ${ɡ}_{m}$ (**Figure 2**). In particular, mesophyll and stomatal conductance share similar responses to abscisic acid (Flexas et al., 2012; Li et al., 2021; Sorrentino et al., 2016), internal CO_2_ concentration (Engineer et al., 2016; Tan et al., 2017), or starch-derived molecules (Lawson et al., 2014), which leads to similar responses under water stress (Flexas et al., 2004; Wang et al., 2018; Xiong et al., 2018). $L_{ɡm}$ became predominant over $L_{ɡs}$ under severe water stress, which was associated with a very low ${ɡ}_{m}$ and a strong restriction of CO_2_ diffusion to chloroplasts (**Figures 2**, **3**). While this partitioning scheme indicated that photosynthesis limitations mostly originated from ${ɡ}_{s}$ , it did not highlight the influence of non-stomatal factors on stomatal conductance. The origins of the decrease in ${ɡ}_{s}$ and $A_{sat}$ can be identified using the USO model equation in the limitation analysis. In particular, the USO partitioning scheme showed that most stomatal closure dynamics can be attributed to a combined effect of ${ɡ}_{m}$ and VPD (**Figure 4**). More specifically, $L_{ɡm,USO}$ was always higher than the other limiting components (**Figures 5A, B**), which highlights the strong control of mesophyll conductance on stomatal closure through its influence on $A_{sat}$ regardless of REW values. These results confirm the importance of the mesophyll constraint for potato, as also highlighted in numerous species across PFTs (Cano et al., 2013; Flexas et al., 2009; Galmés et al., 2007; Grassi and Magnani, 2005; Limousin et al., 2010; Perez-Martin et al., 2014; Wang et al., 2018; Wang et al., 2018; Zait and Schwartz, 2018; Zhu et al., 2021) and emphasize the importance of including the effect of REW on ${ɡ}_{m}$ in LSMs (Knauer et al., 2020). This study also provides a calibration of the water stress factor for potato and contributes to reducing the uncertainties when estimating carbon assimilation and transpiration under water stress (Vidale et al., 2021). Additional information on the description of the physiological effects of mesophyll on stomatal closure can be found in Lemonnier and Lawson (2023). Since disentangling the primary metabolisms that synchronously control photosynthesis, stomatal, and mesophyll conductance remains challenging, future studies would benefit from additional molecular or anatomical measurements to unravel the interplays between stomatal and non-stomatal factors (Gago et al., 2020).

### 4.2 Relationship between photosynthesis and stomatal conductance under severe drought

Severe restrictions in soil water availability induced a decrease in $V_{cmax}$ as well as an increase in ${ɡ}_{1}$ and $C_{i}/C_{s}\;$ (**Figure 2**). An increase in $C_{i}/C_{s}$ can be observed under strong limitations in CO_2_ diffusion and decreasing photosynthetic activity (Bermúdez-Cardona et al., 2015; Brodribb, 1996; Huang, 2020; Tan et al., 2017). In particular, $C_{i}/C_{s}\;$ increased when ${ɡ}_{s}$ was lower than 0.04 mol m^−2^ s^−1^, which was already reported as a stomatal conductance threshold for such $C_{i}$ -inflexion point in various species (Blankenagel et al., 2018; Brodribb, 1996; Flexas, 2002; Martin and Ruiz-Torres, 1992; Rouhi et al., 2007) including potato (Ramírez et al., 2016). In these conditions of photosynthesis inhibition, the excess energy carried by sun irradiance must be metabolized by alternative processes such as xanthophyll (Demmig-Adams et al., 2012), lutein (García-Plazaola et al., 2003), and photorespiratory cycles (Osmond et al., 1980). This last may contribute to the increase in $C_{i}/C_{s}$ by emitting CO_2_ through the glycine decarboxylase enzyme (Busch et al., 2017; Shi and Bloom, 2021).

The increase in ${ɡ}_{1}$ induced an increase in $d{ɡ}_{1}/{ɡ}_{1}$ and $L_{ɡ1}$ when $REW<REW_{th,ɡ1,CiCs}$ (**Figures 2**, **4A, B**). ${ɡ}_{1}$ is inversely related to the marginal carbon cost of water, which corresponds to the change in carbon gained per unit of water transpired, also known as marginal WUE (Medlyn et al., 2011). The increase in ${ɡ}_{1}$ can be explained by either i) an increase in transpiration per unit of carbon gained by photosynthesis or ii) a decrease in photosynthesis per unit of water transpired (Medlyn et al., 2011). For example, increasing stomatal conductance to promote transpiration may help in cooling down leaf surfaces during heatwaves at the expense of increasing mortality risks through hydraulic vulnerability and cavitation (Marchin et al., 2022; Urban et al., 2017). Numerous studies have highlighted such cooling effect on potato (Sprenger et al., 2016; Zhang et al., 2022), which can ultimately lead to an increase in ${ɡ}_{1}$ (Marchin et al., 2023). A decoupling between stomatal conductance and photosynthesis may be the consequence of an adaptive strategy (i.e., sacrificing water for leaf survival and future carbon gains) or the increasing viscosity of water at high temperatures, which facilitates the transport of water in the vascular system (Marchin et al., 2023). In our experiment, the lowest measurement of ${ɡ}_{s}$ was 0.011 mol m^−2^ s^−1^, which is higher than the reported value of minimum stomatal conductance for CO_2_ transfer across plant species (i.e., ${ɡ}_{s}$ = 0.008 mol m^−2^ s^−1^; Duursma et al., 2019) and suggests that stomata may not be fully closed. It is, however, unlikely that potato plants had access to water to sustain transpiration through stomata or cuticles because of the low REW values that were observed in these conditions (**Figure 2**). Alternatively, the increase in ${ɡ}_{1}$ may be caused by a decrease in photosynthesis through the additional effect of NSOL on $A_{sat}$ (Beauclaire et al., 2023; Gourlez de la Motte et al., 2020), which intensifies the decoupling between carbon assimilation and stomatal conductance by decreasing WUE (Manzoni et al., 2011). This hypothesis is supported by previous studies, which have shown that irrigation enhances WUE for potato (Akkamis and Caliskan, 2023; Ati et al., 2012).

The increase in ${ɡ}_{1}$ induced a positive contribution to $\;dA_{sat}/A_{sat}$ (**Figure 5**), suggesting that potato plants promoted the loss of water to the benefit of CO_2_ diffusion despite the risks for the hydraulic and photosynthetic systems when carbon assimilation reached critical levels under drought (Deva et al., 2020; Reynolds-Henne et al., 2010). It indicates a shift in the optimal balance point between carbon gain and water loss where potato plants are willing to lose more water per unit of carbon gained (Zhou et al., 2013). This prioritization is not likely to be driven by optimizing survival under severe drought conditions where soil water is hardly accessible and hydraulic limitations presumably important. Instead, the increase in ${ɡ}_{1}$ could be interpreted as a deviation from optimal stomatal behavior. The stomatal optimality theory states that any increase in the plant’s carbon gain should equal the evaporative water loss proportionally to the carbon cost of water (Cowan and Farquhar, 1977). The optimality theory holds under the assumption that the curvature of photosynthesis versus transpiration is negative; that is, increments of A tend to become smaller with increments of ${ɡ}_{s}$ , as stomata reduce the gradient for CO_2_ uptake more than that for H_2_O loss (Buckley et al., 2017). Any conditions shifting the curve to a positive curvature will cause a deviation from the optimality theory, challenging the interpretation of ${ɡ}_{1}$ short-term dynamics. Two of these conditions were likely observed in this study: first, an additional restriction of CO_2_ diffusion to chloroplasts by mesophyll conductance and, second, a possible hydraulic impairment at very low REW, which ultimately changes the photosynthesis–transpiration relationship (Buckley et al., 2017; Cowan and Farquhar, 1977). This unrealistic stomatal opening response is consistent with previous studies that have shown a similar increase in ${ɡ}_{1}$ under severe drought (Beauclaire et al., 2023; Gourlez de la Motte et al., 2020; Zhou et al., 2013), arguing for a refinement of stomatal optimality. Novel modeling approaches consider the cost of stomatal opening as a function of an increase in NSOL (Dewar et al., 2018), or hydraulic impairment using profit maximization optimization (Sperry et al., 2017) may be preferred to interpret stomatal dynamics under drought conditions.

### 4.3 Methodological considerations

${ɡ}_{m}$ was determined by the “variable J” method at light saturation (Harley et al., 1992), which is sensitive to variation in $R_{d}$ and $\Gamma^{*}$ (Pons et al., 2009; Théroux-Rancourt et al., 2014). These two variables can be impacted by drought and heat stress, which was not considered in the method part. First, it has been shown that $R_{d}$ can increase under water stress due to the additional release of CO_2_ from mitochondria by the photorespiratory cycle (Busch et al., 2017; Pinheiro and Chaves, 2011). Second, the sensitivity of $\Gamma^{*}$ to temperature can change under critical levels (usually above 30°C), which may invalidate the parameterization on leaf temperature (Bernacchi et al., 2001). Measuring the CO_2_ compensation point (Walker and Ort, 2015) and leaf respiration (Yin and Amthor, 2024) under drought could help resolve these uncertainties.

The diffusion of water vapor through the cuticle and epidermis may become significant compared to stomatal diffusion under water stress (Boyer, 2015a; Boyer, 2015b; Boyer et al., 1997). As the transpiration flux measured by gas exchange measurement systems corresponds to the sum of the diffusion through stomatal and cuticle conductance, $C_{i}$ overestimations can occur as the Fick law considers an identical gas phase path for CO_2_ and H_2_O. Direct measurements of $C_{i}$ by a modified gas exchange device (Boyer and Kawamitsu, 2011) or a modification of the Fick law by quantifying the cuticle conductance (Wang et al., 2018) could increase the accuracy of $C_{i}$ under water stress.

Lastly, none of the current methods for estimating ${ɡ}_{m}\;$ actually measure diffusion plant conductance. This paper interprets ${ɡ}_{m}$ as an internal diffusion plant conductance limiting CO_2_ diffusion from substomatal cavities to carboxylation sites in the chloroplasts. This two-dimensional view of CO_2_ diffusion is a simplification of the actual pathway where sink and sources are distributed along the way. The widely adopted definition of mesophyll conductance (i.e., $A/\left(C_{i}-C_{c}\right)$ ) simplifies the leaf as a single sink and ignores the complexity of the mesophyll structure, as well as the heterogeneity in photosynthetic capacities and cellular structure of the leaf vertical light absorption profile (Evans et al., 2009). A more realistic view of mesophyll conductance should include i) a decomposition of resistive components on the CO_2_ pathway such as cell wall and membrane, cytosol, chloroplast envelope, and stroma resistances (Cousins et al., 2020); ii) three-dimensional modeling across the leaf vertical profile (Théroux-Rancourt and Gilbert, 2017; Xiao and Zhu, 2017); and iii) a quantification of chloroplast movement, which is a key driver of ${ɡ}_{m}$ (Carriquí et al., 2019) and is sensitive to changes in light absorption peaks (Tholen et al., 2008). However, most of the complexity can be, neglected when measurements are conducted at light saturation (Théroux-Rancourt and Gilbert, 2017). Improvements in the techniques for estimating the contribution of the different resistive components would help in understanding the response of ${ɡ}_{m}$ to anatomical and biochemical drivers under drought (Evans, 2021).

## Data Availability

The raw data supporting the conclusions of this article will be made available by the authors, without undue reservation.
