# Supplementary material for: Key role played by mesophyll conductance in limiting carbon assimilation and transpiration of potato under soil water stress
Source: Front Plant Sci. 2024 Dec 2;15:1500624. doi: 10.3389/fpls.2024.1500624 (PMC11664552; doi:10.3389/fpls.2024.1500624)
Supplement: Supplementary file 1 [file DataSheet1.docx]

***Supplementary Material***

**Supplementary Method :
Mathematical development of the partitioning scheme using the USO and FvCB models**

The mathematical development of the limitation analysis using the unified stomatal optimality (USO) model (Medlyn et al., 2011) and the Farquhar, von-Caemmerer, Berry (FvCB) model (Farquhar et al., 1980) can be demonstrated by starting from the equation describing the limitation of carbon assimilation under high irradiance ($A_{sat}$) by Rubisco as:

$$\begin{aligned} A_{sat}=V_{cmax}\frac{C_{c}-\Gamma^{*}}{C_{c}+K_{m}} \#\left( S1 \right) \end{aligned}$$

where $V_{cmax}$ is the maximum carboxylation rate of Rubisco, $C_{c}$the CO_2_ concentration in the chloroplast, $\Gamma^{*}$the CO_2_ compensation point, $K_{m}$ the Michaelis-Menten coefficient of Rubisco kinetics. At 25°C (i.e., no effects of temperature on model parameters) and neglecting leaf respiration, the total derivative of $A_{sat}$ is:

$$\begin{aligned} dA_{sat}=\frac{\delta A_{sat}}{\delta V_{cmax}}dV_{cmax}+\frac{\delta A_{sat}}{\delta C_{c}}dC_{c} \#(S2) \end{aligned}$$

with $\frac{\delta A_{sat}}{\delta V_{cmax}}=\frac{A_{sat}}{V_{cmax}}$ and $\frac{\delta A_{sat}}{\delta C_{c}}=V_{cmax}\frac{K_{m}+\Gamma^{*}}{\left( C_{c}+K_{m} \right)^{2}}$. The total derivative of $C_{c}$ can be determined using the Fick law (Farquhar and Sharkey, 1982) as:

$$\begin{aligned} C_{c}=C_{s}-\frac{A_{sat}}{g_{s}}-\frac{A_{sat}}{g_{m}} \#\left( S3 \right) \end{aligned}$$

with $C_{s}$ the CO_2_ concentration at the leaf surface, $g_{s}$ and $g_{m}$the stomatal and mesophyll conductance to CO_2_ transfer. When the dependence of $g_{s}$to vapor pressure deficit (VPD), CO_2_ concentration and carbon assimilation is not considered (i.e., the USO model not implemented in the limitation analysis), the total derivative of $C_{c}$ is:

$$\begin{aligned} {dC}_{c}=\frac{\delta C_{c}}{\delta A_{sat}}dA_{sat}+\frac{\delta C_{c}}{\delta g_{s}}dg_{s}+\frac{\delta C_{c}}{\delta g_{m}}dg_{m} \#\left( S4 \right) \end{aligned}$$

$$\begin{aligned} {dC}_{c}=\left( -\frac{1}{g_{m}}-\frac{1}{g_{s}} \right)dA_{sat}+\frac{A_{sat}}{{g_{s}}^{2}}dg_{s}+\frac{A_{sat}}{{g_{m}}^{2}}dg_{m} \#\left( S5 \right) \end{aligned}$$

Combining Eq. S5 and Eq. S2 gives:

$$\begin{aligned} dA_{sat}=\frac{A_{sat}}{V_{cmax}}dV_{cmax}+\frac{\delta A_{sat}}{\delta C_{c}}\left[ \left( -\frac{1}{g_{m}}-\frac{1}{g_{s}} \right)dA_{sat}+\frac{A_{sat}}{{g_{s}}^{2}}dg_{s}+\frac{A_{sat}}{{g_{m}}^{2}}dg_{m} \right]\#\left( S6 \right) \end{aligned}$$

Simplifying Eq. S6 and writing $dA_{sat}$ on the left-hand side gives:

$$\begin{aligned} dA_{sat}+\frac{\delta A_{sat}}{\delta C_{c}}\left( \frac{1}{g_{m}}+\frac{1}{g_{s}} \right)dA_{sat}=\frac{A_{sat}}{V_{cmax}}dV_{cmax}+\frac{\delta A_{sat}}{\delta C_{c}}\frac{A_{sat}}{{g_{s}}^{2}}dg_{s}+\frac{\delta A_{sat}}{\delta C_{c}}\frac{A_{sat}}{{g_{m}}^{2}}dg_{m}\#\left( S7 \right) \end{aligned}$$

$$\begin{aligned} dA_{sat}=\frac{A_{sat}\frac{dV_{cmax}}{V_{cmax}}}{1+\frac{\delta A_{sat}}{\delta C_{c}}\left( \frac{1}{g_{m}}+\frac{1}{g_{s}} \right)}+\frac{\frac{\delta A_{sat}}{\delta C_{c}}\frac{A_{sat}}{g_{s}}\frac{dg_{s}}{g_{s}}}{1+\frac{\delta A_{sat}}{\delta C_{c}}\left( \frac{1}{g_{m}}+\frac{1}{g_{s}} \right)}+\frac{\frac{\delta A_{sat}}{\delta C_{c}}\frac{A_{sat}}{g_{m}}\frac{dg_{m}}{g_{m}}}{1+\frac{\delta A_{sat}}{\delta C_{c}}\left( \frac{1}{g_{m}}+\frac{1}{g_{s}} \right)} \#\left( S8 \right) \end{aligned}$$

Finally, simplifying Eq. S8 in terms of relative changes in $A_{sat}$ and implementing the total conductance to CO_2_ transfer $g_{t}=\left( \frac{1}{gs}+\frac{1}{gm} \right)^{-1}$gives:

$$\begin{aligned} \frac{dA_{sat}}{A_{sat}}=\frac{g_{t}\frac{dV_{cmax}}{V_{cmax}}}{g_{t}+\frac{\delta A_{sat}}{\delta C_{c}}}+\frac{\frac{\delta A_{sat}}{\delta C_{c}}\frac{g_{t}}{g_{s}}\frac{dg_{s}}{g_{s}}}{g_{t}+\frac{\delta A_{sat}}{\delta C_{c}}}+\frac{\frac{\delta A_{sat}}{\delta C_{c}}\frac{g_{t}}{g_{m}}\frac{dg_{m}}{g_{m}}}{g_{t}+\frac{\delta A_{sat}}{\delta C_{c}}}\#\left( S9 \right) \end{aligned}$$

$$\begin{aligned} \frac{dA_{sat}}{A_{sat}}=\frac{dg_{s}}{g_{s}}l_{gs}+\frac{dg_{m}}{g_{m}}l_{gm}+\frac{dV_{cmax}}{V_{cmax}}l_{Vcmax}=L_{gs}+L_{gm}+L_{Vcmax} \#\left( S10 \right) \end{aligned}$$

with:

$$\begin{aligned} l_{gs}=\frac{\frac{g_{t}}{g_{s}}\frac{\delta A_{sat}}{\delta C_{c}}}{g_{t}+\frac{\delta A_{sat}}{\delta C_{c}}} \#\left( S11 \right) \end{aligned}$$

$$\begin{aligned} l_{gm}=\frac{\frac{g_{t}}{g_{m}}\frac{\delta A_{sat}}{\delta C_{c}}}{g_{t}+\frac{\delta A_{sat}}{\delta C_{c}}} \#\left( S12 \right) \end{aligned}$$

$$\begin{aligned} l_{Vcmax}=\frac{g_{t}}{g_{t}+\frac{\delta A_{sat}}{\delta C_{c}}} \#\left( S13 \right) \end{aligned}$$

where $L$ is the limitation component and $l$the relative limitation component. Eq. S10 is the limitation analysis scheme described in Grassi and Magnani (2005).

The second partitioning scheme considered in this study is obtained by implementing the USO model (Eq. S14) for calculating the total derivative of $g_{s}$($dg_{s},$Eq. S10) as:

$$\begin{aligned} g_{s}=\left( 1+\frac{g_{1}}{\sqrt{VPD}} \right)\frac{A_{sat}}{C_{s}}\#\left( S14 \right) \end{aligned}$$

$$\begin{aligned} {dg}_{s}=\frac{\delta g_{s}}{\delta g_{1}}dg_{1}+\frac{\delta g_{s}}{\delta A_{sat}}dA_{sat}+\frac{\delta g_{s}}{\delta VPD}dVPD +\frac{\delta g_{s}}{\delta C_{s}}dC_{s}\#\left( S15 \right) \end{aligned}$$

Calculating the partial derivatives of each term and considering $C_{s}$ as constant gives:

$$\begin{aligned} {dg}_{s}=\frac{A_{sat}}{C_{s}\sqrt{VPD}}dg_{1}+\frac{\left( 1+\frac{g_{1}}{\sqrt{VPD}} \right)}{C_{s}}dA_{sat}-\frac{1}{2}\frac{A_{sat} g_{1}}{C_{s}{VPD}^{\frac{3}{2}}}dVPD \#\left( S16 \right) \end{aligned}$$

Writing Eq. S16 in terms of relative changes in $g_{s}$ gives:

$$\begin{aligned} \frac{{dg}_{s}}{g_{s}}=\left( \frac{g_{1}}{g_{1}+\sqrt{VPD}} \right)\frac{dg_{1}}{g_{1}}+\frac{dA_{sat}}{A_{sat}}-\frac{1}{2}\left( \frac{g_{1}}{g_{1}+\sqrt{VPD}} \right)\frac{dVPD}{VPD} \#\left( S17 \right) \end{aligned}$$

Eq. S17 gives the relative variation of $g_{s}$ with respect to the relative variations of $g_{1}$, $A_{sat}$ and $VPD$. Moreover, it can be shown that $\frac{g_{1}}{g_{1}+\sqrt{VPD}}=\frac{C_{i}}{C_{s}}$:

$$\begin{aligned} \frac{C_{i}}{C_{s}}=1-\frac{\sqrt{VPD}}{\sqrt{VPD}+g_{1}}=\frac{g_{1}}{g_{1}+\sqrt{VPD}} \#\left( S18 \right) \end{aligned}$$

which gives, after combining Eq. S18 into Eq. S17:

$$\begin{aligned} \frac{{dg}_{s}}{g_{s}}=\frac{C_{i}}{C_{s}}\frac{dg_{1}}{g_{1}}+\frac{dA_{sat}}{A_{sat}}-\frac{1}{2}\frac{C_{i}}{C_{s}}\frac{dVPD}{VPD}\#\left( S19 \right) \end{aligned}$$

Finally, combining Eq. S19 and Eq. S10 gives, after simplification:

$$\begin{aligned} \frac{dA_{sat}}{A_{sat}}=\frac{dg_{1}}{g_{1}}\left( \frac{l_{gs}}{1-l_{gs}}\frac{C_{i}}{C_{s}} \right)+\frac{dg_{m}}{g_{m}}\left( \frac{l_{gm}}{1-l_{gs}} \right)+\frac{dV_{cmax}}{V_{cmax}}\left( \frac{l_{Vcmax}}{1-l_{gs}} \right)-\left( \frac{1}{2}\frac{l_{gs}}{1-l_{gs}}\frac{C_{i}}{C_{s}} \right)\frac{dVPD}{VPD} \#\left( S20 \right) \end{aligned}$$

$$\begin{aligned} \frac{dA_{sat}}{A_{sat}}=\frac{dg_{1}}{g_{1}}l_{g1,USO}+\frac{dg_{m}}{g_{m}}l_{gm,USO}+\frac{dV_{cmax}}{V_{cmax}}l_{Vcmax,USO}+\frac{dVPD}{VPD} l_{VPD,USO}\#\left( S21 \right) \end{aligned}$$

$$\begin{aligned} \frac{dA_{sat}}{A_{sat}}=L_{g1,USO}+L_{gm,USO}+L_{Vcmax,USO}+L_{VPD,USO} \#\left( S22 \right) \end{aligned}$$

Eq. S21 and Eq. S22 link the relative variation of $A_{sat}$ to the relative variations of $VPD$, $g_{1}$, $g_{m}$ and $V_{cmax}$ (Farquhar et al., 1980; Medlyn et al., 2011).

**Supplementary Figures and Tables**

**Supplementary Table 1.** Akaike information criterion corrected for small sample size (AICc) of the linear and linear-plateau models describing the response of $A_{sat}$, $V_{cmax,25}$, $g_{s}$, $g_{m25}$, $g_{1}$ and $C_{i}/C_{s}$ to REW. ΔAICc is the difference between the lowest AICc and the linear or segmented model for each variable. A ΔAICc of 0 indicates the corresponding model had the lowest AICc.

| ΔAICc= AICc-min(AICc) | ΔAICc Linear model | ΔAICc Segmented model |
| --- | --- | --- |
| $\boldsymbol{A}_{\boldsymbol{sat}}$ | 0 | -3.36 |
| $\boldsymbol{V}_{\boldsymbol{cmax,25}}$ | -7.45 | 0 |
| $\boldsymbol{g}_{\boldsymbol{s}}$ | 0 | 4.72 |
| $\boldsymbol{g}_{\boldsymbol{m,25}}$ | 0 | 5.67 |
| $\boldsymbol{g}_{\boldsymbol{1}}$ | 23 | 0 |
| $\boldsymbol{C}_{\boldsymbol{i}}\boldsymbol{/}\boldsymbol{C}_{\boldsymbol{s}}$ | 6.22 | 0 |


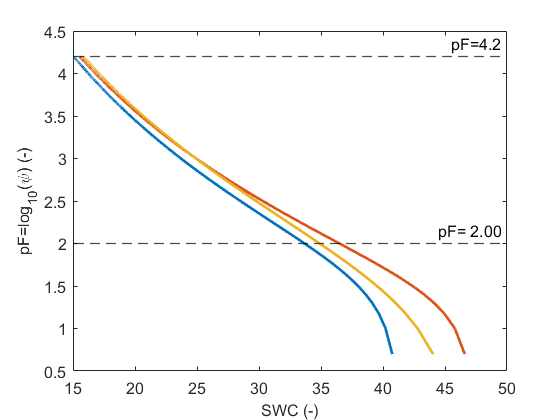


**Figure S1.** Soil water retention curves (SWRC) of the three soil samples collected at 15 cm depth. The field capacity and wilting point were calculated as the average values of soil water content (SWC) at a pF (log $\psi$, with $\psi$the suction head) of respectively 2 and 4.2. SWRC were obtained from the van Genuchten model (van Genuchten, 1980) : $SWC={SWC}_{r}+\frac{{SWC}_{r}-{SWC}_{s}}{\left| 1+\left( \alpha\left| \psi\right|^{n} \right) \right|^{m}}$ with${SWC}_{r}$the residual SWC, ${SWC}_{s}$ the saturated SWC, $\psi$ the suction head, $\alpha$ related to the inverse of the air entry suction, $n$ and $m$ are empirical parameters. The parameters of the three SWRC are ${SWC}_{s}$=0.412, ${SWC}_{r}$=0, $\alpha$=0.028, $n$=1.27, $m$=0.13 (blue curve), ${SWC}_{s}$=0.471, ${SWC}_{r}$=0, $\alpha$=0.04, $n$=1.45, $m$=0.118 (orange curve), and ${SWC}_{s}$=0.460, ${SWC}_{r}$=0, $\alpha$=0.037, $n$=0.84, $m$=0.20 (yellow curve).


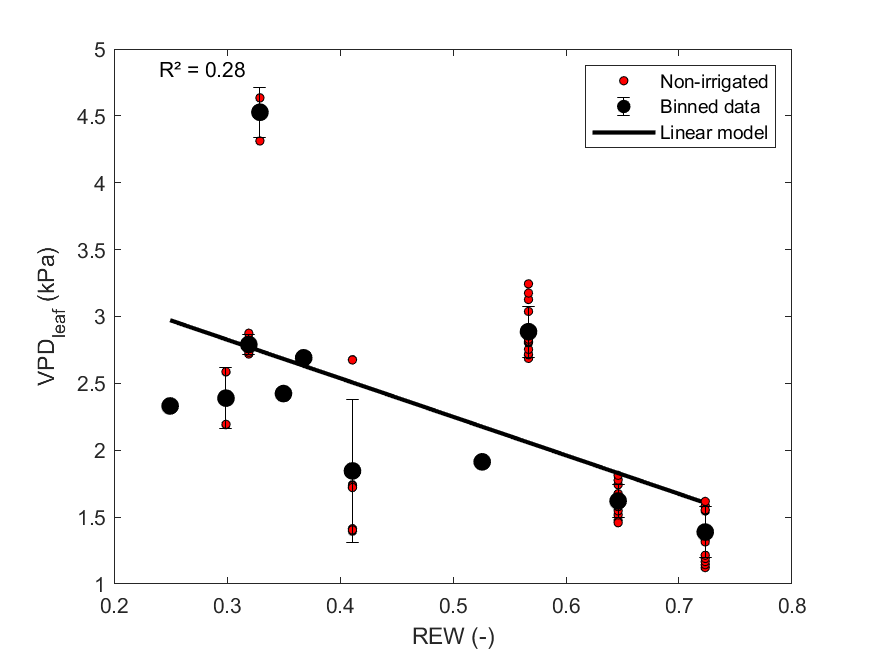


**Figure S2.** Relationship between vapor pressure deficit at the leaf surface (VPDleaf) and relative extractable water (REW) for non-irrigated plants.


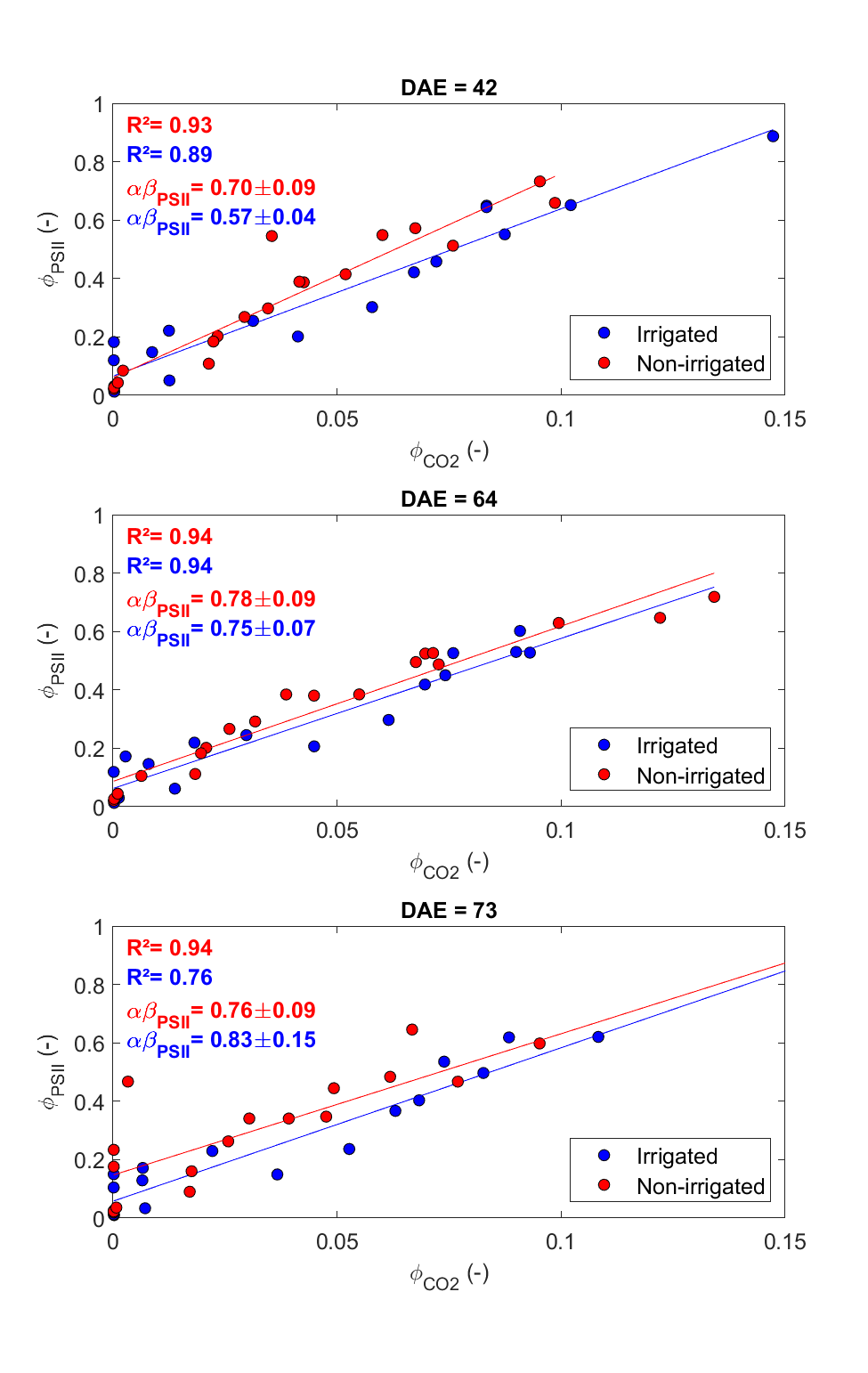


**Figure S3.** Relationship between the fluorescence quantum yield of PSII ($\phi_{PSII}$) and the apparent quantum yield of CO_2_ assimilation ($\phi_{CO2}$) for three different days after emergence (DAE) during the drought experiment. For each DAE, a linear regression ($\phi_{PSII}=k \phi_{CO2}+k'$) was fitted for irrigated (blue) and non-irrigated (red) data and the product of leaf absorptance to the fraction of the absorbed PAR allocated to PSII ($\alpha\cdot\beta_{PSII}$) was calculated as 4/$k$.

**References**

Farquhar, G.D., von Caemmerer, S., Berry, J.A., 1980. A biochemical model of photosynthetic CO2 assimilation in leaves of C3 species. Planta 149, 78–90. https://doi.org/10.1007/BF00386231

Grassi, G., Magnani, F., 2005. Stomatal, mesophyll conductance and biochemical limitations to photosynthesis as affected by drought and leaf ontogeny in ash and oak trees. Plant Cell Environ. 28, 834–849. https://doi.org/10.1111/j.1365-3040.2005.01333.x

Medlyn, B.E., Duursma, R.A., Eamus, D., Ellsworth, D.S., Prentice, I.C., Barton, C.V.M., Crous, K.Y., De Angelis, P., Freeman, M., Wingate, L., 2011. Reconciling the optimal and empirical approaches to modelling stomatal conductance: RECONCILING OPTIMAL AND EMPIRICAL STOMATAL MODELS. Glob. Change Biol. 17, 2134–2144. https://doi.org/10.1111/j.1365-2486.2010.02375.x
